# The FOXD1/NAT10 positive feedback loop drives nasopharyngeal carcinoma progression

**DOI:** 10.1186/s41065-025-00555-9

**Published:** 2025-09-25

**Authors:** Leifeng Liu, Qizhu Chen, Yiling Li, Weihao Wu, Feng Jiang, Haitao Qiu

**Affiliations:** Department of Otorhinolaryngology Head and Neck Surgery, Affiliated Hospital of Guangdong Medical University, No. 57, South Renmin Avenue, Xiashan District, Zhanjiang, 524000 Guangdong China

**Keywords:** Nasopharyngeal carcinoma, FOXD1, Transcription factor, ac4C modification, NAT10

## Abstract

**Background:**

Nasopharyngeal carcinoma (NPC) is a malignant epithelial tumor. N4-acetylcytidine (ac4C) modification regulates mRNA stability and contributes to tumorigenesis. FOXD1, a crucial transcription factor, acts as a tumor-promoting factor in NPC. However, its molecular underpinnings are not fully elucidated.

**Methods:**

Expression of mRNA and protein was quantified by quantitative PCR, immunohistochemistry, or immunoblotting. The effects on cell phenotypes were determined by assessing NPC cell proliferation, apoptosis, invasiveness, sphere formation, and TUVEC tube formation. The interaction between FOXD1 and N-acetyltransferase 10 (NAT10) was predicted by online computational methods and validated using RIP, mRNA stability, ChIP, and luciferase assays. Xenograft studies were performed to observe the in vivo effects.

**Results:**

FOXD1 expression was increased in NPC clinical samples and cell lines. Functionally, FOXD1 depletion suppressed NPC cell growth, invasion, sphere formation ability, while promoting cell apoptosis and impairing HUVEC tube formation. Mechanistically, NAT10 stabilized FOXD1 mRNA by mediating its ac4C modification. FOXD1 upregulation counteracted NAT10 depletion-driven cellular phenotypic alterations. In turn, FOXD1 acted as a transcriptional activator of NAT10 in NPC cells. NAT10 reconstitution reversed FOXD1 depletion-driven cellular phenotypic alterations. Additionally, NAT10 knockdown inhibited NPC xenograft growth in vivo by reducing FOXD1 levels.

**Conclusion:**

Our study demonstrates that a mutually reinforcing FOXD1/NAT10 positive feedback loop drives NPC progression, providing new therapeutic vulnerabilities for clinical intervention.

**Supplementary Information:**

The online version contains supplementary material available at 10.1186/s41065-025-00555-9.

## Introduction

Nasopharyngeal carcinoma (NPC), a malignant epithelial tumor originating from the nasopharynx, exhibits distinct geographical predilection with high prevalence in North Africa, Southeast Asia, and Southern China [1, 2]. NPC development is strongly linked to Epstein-Barr virus (EBV) infection, genetic susceptibility, environmental carcinogens, and chronic inflammatory stimuli [1]. Current standard therapies for NPC primarily include radiotherapy and platinum-based chemotherapy, achieving 5-year survival rates of 70–80% for early-stage disease. However, recurrence and distant metastasis persist in 20–30% of cases, compounded by radioresistance and chemotherapy-induced toxicity [3, 4]. Emerging strategies, such as PD-1/PD-L1 inhibitors, show promise in recurrent or metastatic NPC [5], but heterogeneous treatment responses and acquired resistance underscore the need for biomarkers to guide personalized treatment. Emerging evidence implicates that dysregulation of epigenetic machinery, particularly mRNA modification pathways, constitutes a key driver of NPC pathogenesis by promoting oncogenic transformation and cancer progression [6, 7]. Unraveling these molecular intricacies will pave the way for precision therapies to overcome treatment resistance and improve clinical outcomes.

FOXD1, a member of the forkhead box (FOX) transcription factor family, governs embryonic development, cell cycle regulation, and metabolic homeostasis by controlling target gene expression [8]. In oncogenesis, FOXD1 exhibits oncogenic properties across multiple cancers by modulating EMT, stemness, and therapy resistance through diverse signaling pathways [9]. For example, it promotes growth, invasion, and metastasis in pancreatic cancer cells by enhancing aerobic glycolysis via dual regulation of SLC2A1, leading to GLUT1 overexpression [10]. FOXD1 stabilization correlates with enhanced tumorigenicity of mesenchymal stem cells and poor survival of glioblastoma patients [11]. FOXD1 promotes basal-like breast cancer progression by maintaining tumor-promoting transcriptional networks [12]. In NPC, FOXD1 has been shown to contribute to cancer progression and gemcitabine resistance by enhancing mitophagy [13]. However, the molecular underpinnings of its oncogenic action not are fully elucidated.

N4-acetylcytidine (ac4C) modification, a newly identified mRNA modification, controls mRNA stability and translational efficiency [14]. Emerging studies highlight the implication of dysregulated ac4C modification in cancer progression, where hyperacetylated oncogenic transcripts drive proliferation, metastasis, and chemoresistance [15, 16]. N-acetyltransferase 10 (NAT10), as the only known ac4C “writer”, catalyzes ac4C modification in eukaryotic RNA, thereby influencing mRNA stability and regulating disease development [17]. Recent studies have shown its pro-tumorigenic function in multiple cancers, such as bladder and gastric cancers, by mediating mRNA ac4C modification and rewiring cancer cell metabolism and epigenetics [16, 18]. Furthermore, NAT10 has been identified as a tumor-promoting factor in NPC through its regulation in mRNA ac4C modification and stability [19]. Despite these advances, the interplay between NAT10 and FOXD1 in NPC pathogenesis remains unexplored.

The current study addressed these questions by combining bioinformatics analyses and experimental validation, elucidating a novel mechanism by which a FOXD1/NAT10 positive feedback loop drives NPC progression via coordinated FOXD1 mRNA acetylation and NAT10 transcriptional reprogramming. Our findings provide novel molecular insights into mechanisms of NPC progression, thereby pointing to biomarkers and intervention possibilities.

## Materials and methods

### Patient samples

This study utilized surgically resected tumor specimens (*n* = 36) and adjacent non-tumor nasopharyngeal epithelial tissues (*n* = 36) collected from NPC patients undergoing surgery in the Affiliated Hospital of Guangdong Medical University. These samples were used for evaluation of FOXD1 expression. All samples were acquired with informed consent from all subjects in accordance with approval by the institution’s Ethics Committee of Affiliated Hospital of Guangdong Medical University.

### Cell lines and culture conditions

The study employed four human NPC cell lines: HK1 (#CL-0695, Procell, Wuhan, China), SNU46 (#YS0071C, YaJi Biological, Shanghai, China), HNE3 (#YY-CS-0368, Y-Y Chemical, Shanghai, China), and C666-1 (#IM-H432, Immocell, Xiamen, China). Human nasopharyngeal epithelial cell line NP69 (#IM-H435, Immocell) served as the control, and HUVECs (#IM-H205, Immocell) were utilized in tube formation assay. Four NPC cell lines were cultured in RPMI-1640 (HyClone, Logan, UT, USA) containing 1% penicillin/streptomycin (Servicebio, Wuhan, China) and 10% FBS (Nichirei Bioscience, Tokyo, Japan). NP69 and HUVEC cell lines were grown using Immocell-provided specialized standard mediums (#IM-H435-1 and #IM-H205-1). All cell cultures were maintained at 37 °C in a humidified atmosphere of 5% CO_2_.

### Constructs, transient transfection, and viral infections

For gene silencing experiments, siRNA pools (MCE, Shanghai, China): FOXD1 Human Pre-designed siRNA Set A (si-FOXD1), si-FOXD1#2, and NAT10 Human Pre-designed siRNA Set A (si-NAT10) were employed. Each set comprised three specific siRNA duplexes along with corresponding scrambled controls (si-NC). For functional rescue studies, pLV3-CMV-FOXD1(human)-Puro (OE-FOXD1) and pLV3-CMV-NAT10(human)-Neo (OE-NAT10) were obtained from Miaoling Plasmid (Wuhan, China), with OE-NC as the matched control construct vector. HNE3 and C666-1 cell lines (2 × 10^4^ cells/well) were plated in 24-well plates and allowed to adhere for 24 h. Subsequent transfection was carried out with Lipofectamine 3000 reagent (Invitrogen, Saint-Aubin, France), administering either 50 nM siRNA, 500 ng plasmid DNA, or both. Following incubation periods of 24–72 h, transfected cell populations were harvested for follow-up studies.

For the establishment of a stable NAT10 depletion cell line, lentiviral vectors encoding NAT10-targeting shRNA (sh-NAT10) and scrambled control shRNA (sh-NC) were custom-produced by VectorBuilder (Guangzhou, China). Following reference protocols [20], C666-1 cell cultures were transduced with viral particles in media containing 8 µg/mL polybrene. To obtain the stable cell line, puromycin selection (2 µg/mL) began 20–24 h post-infection and continued for at least 14 days.

### FOXD1 and NAT10 mRNA expression by quantitative PCR

For RNA extraction, either clinical specimens or transfected NPC cells were processed with Trizol-based isolation (Invitrogen). Following purification, cDNA synthesis was performed through reverse transcription using IScript Kit (Bio-Rad, Glattbrugg, Switzerland). Gene expression levels were quantified using TB Green Premix Ex Taq II (TaKaRa, Dalian, China) on a Real-time System (CFX Connect, Bio-Rad), with the primers of FOXD1 (forward: 5’-AGAAGGTAGGAATCCCGGCT-3’, reverse: 5’-CCGGGCTGTTGACAGTTTTG-3’) and NAT10 (forward: 5’-TTTCGGAGTTGTTCCGTGCT-3’, reverse: 5’-CTTCCGGTGACTGCGCC-3’). Transcripts of GAPDH (5’-AATGGGCAGCCGTTAGGAAA-3’-forward and 5’-GCGCCCAATACGACCAAATC-3’-reverse) were used for normalization. 2^−ΔΔCt^ quantification method was used for quantitative PCR analyses.

### Xenograft studies

All procedures involving animals were carried out in accordance with National Ethical Guidelines and were approved by the Institutional Animal Care Committee of Affiliated Hospital of Guangdong Medical University, using 6-week-old female BALB/c nude mice, which were obtained from Sibeifu Biotechnology Co., Ltd. (Beijing, China) and grouped into three groups: sh-NC (*n* = 5), sh-NAT10 (*n* = 5), and sh-NAT10 + OE-FOXD1 (*n* = 5). For xenograft induction, nude mice received subcutaneous injections of NAT10-depleted C666-1 cells or sh-NC controls in the right flank (2 × 10^6^ cells/mouse in 150 µL of PBS). Tumor diameters were measured every five days using calipers, with volume calculated as (width)^2^ × length × 0.5. Intratumoral injections of OE-FOXD1 constructs (2 µg per mouse) were administered upon reaching a tumor volume of ~ 100 mm^3^. The study duration was 29 days post-implantation, after which xenografts were excised, measured, and documented photographically.

### Histology and immunohistochemical staining

After 24-hour fixation in 10% neutral-buffered formalin, xenograft tumors underwent standard processing including paraffin embedding and microtome sectioning (4 μm) prior to histology staining with hematoxylin and eosin (H&E). Tumor sections underwent immunohistochemical staining for Ki67 and FOXD1 detection. Following standard rehydration and antigen retrieval procedures, samples were blocked with 2% BSA prior to overnight incubation with primary antibodies (rabbit polyclonal) to Ki67 (#27309-1-AP, Proteintech, Wuhan, China, 1:6,000), NAT10 (#13365-1-AP, Proteintech, 1:10,000), CD34 (#14486-1-AP, Proteintech, 1:3,000), or FOXD1 (#PA5-35145, Invitrogen, 1:50). Signal detection was achieved using HRP secondary antibody followed by DAB chromogenic development (DAB Kit, Beyotime, Shanghai, China).

### Antibodies and immunoblotting

Following extraction from clinical specimens or cultured cells, proteins (20 µg) were resolved on 12% SDS-PAGE and immunoblotted onto PVDF membranes (Beyotime) for immunoblotting. Following blocking, membranes were probed overnight at 4 °C with primary antibodies against FOXD1 (#PA5-35145, rabbit polyclonal, Invitrogen, 1:2,000), NAT10 (#ab194297, rabbit monoclonal, Abcam, Cambridge, UK, 1:2,000), and GAPDH (#ab181602, rabbit monoclonal, Abcam, 1:10,000). After incubation with HRP anti-rabbit secondary antibody, bands were developed using enhanced chemiluminescence technique (ChemiDoc XRS System, Bio-Rad) and detected by LAS4000 Mini imager (Fuji Film, Minamiashigara, Japan), followed by quantitative analysis of band intensities using ImageJ (NIH, Bethesda, MD, USA).

### EdU proliferation assay

DNA synthesis activity for proliferation analysis was evaluated through EdU incorporation assays performed with Cell-Light Apollo488 Kit (Ribobio, Guangzhou, China), adhering strictly to the provided technical protocols. Forty-eight hours following transfection, HNE3 and C666-1 cell cultures were exposed to 10 µM EdU labeling solution. After fixation, nuclear DNA synthesis was visualized through dual fluorescence staining: Apollo488 (green) identified proliferating cells while DAPI (blue) marked all nuclei. Quantitative analysis of cell proliferation rates was performed by calculating the percentage of EdU-positive cells using Leica SP8 confocal microscopy (Leica, Wetzlar, Germany).

### Flow cytometry

Seventy-two hours after transfection, cellular apoptosis (HNE3 and C666-1) was evaluated using dual staining with FITC-conjugated Annexin V and propidium iodide (PI) (Apoptosis Detection Kit, BD Biosciences, Cowley, UK). Cell cycle distribution analysis was performed using a PI-staining Cell Cycle Kit (Beyotime). Cell populations were subsequently analyzed by flow cytometry (BD LSRII system, BD Biosciences).

### Transwell assay for cell invasion

After 24 h of transfection, 1 × 10^5^ cells HNE3 and C666-1 cells were resuspended in non-serum medium. Cell suspensions were loaded into Transwell chambers (8-µm pore size; Corning, Shanghai, China) positioned in 24-well plates containing complete medium. Following a 24-hour incubation period, the cells that had passed through the pores were subjected to crystal violet (0.5%) staining and subsequent counting using the Leica DM2500 microscope.

### Sphere formation assay

Post-transfection for 24 h, HNE3 and C666-1 cells were maintained in serum-free medium for 24 h prior to plating in Corning Ultra-Low Attachment 96-well plates (Corning) for sphere formation assays. Following 7 days of culture under standard conditions (37 °C, 5% CO_2_), spheres were quantified using a Leica DM2500 microscope to assess clonogenic potential.

### Tube formation assay

After 48 h transfection, the conditioned medium from transfected HNE3 and C666-1 cells was collected and used to assess the impact on HUVEC tube formation ability. HUVEC suspensions (1 × 10^5^ cells/well) prepared in equal parts conditioned and standard media were transferred to 48-well plates pre-coated with Matrigel. Tubular network formation was quantified at 6 h post-incubation using ImageJ analysis of five representative fields per well [21]. Relative tube formation rate in experimental group was calculated relative to the control group.

### Bioinformatics analysis

To identify potential ac4C modification sites in FOXD1 mRNA, the PACES web-based platform (http://www.rnanut.net/paces/) was utilized. The interaction between NAT10 and FOXD1 mRNA was computationally predicted using the RPISeq algorithm (http://pridb.gdcb.iastate.edu/RPISeq/#). Additionally, the JASPAR database (https://jaspar.elixir.no/) was employed to analyze potential binding sites of FOXD1 within the NAT10 promoter region (-2000 bp to TSS).

### Ac4C RIP-quantitative PCR

The ac4C RIP experiment was conducted according to the protocols provided with the GenSeq ac4C RIP Kit (Cloudseq Biotech, Shanghai, China). In brief, 200 µg of total RNA from HNE3 and C666-1 cells was fragmented into 100–200 bp segments. Subsequently, 5 µg of either anti-ac4C antibody or anti-IgG isotype control was mixed with magnetic beads and then incubated with the fragmented RNA. Following immunoprecipitation, the RNA was isolated and purified, after which quantitative PCR was employed to assess the enrichment levels of FOXD1 mRNA fragments.

### Analysis of FOXD1 mRNA stability

Following transfection with si-NAT10 or si-NC, HNE3 and C666-1 cells were exposed to 5 µg/mL Actinomycin D (Act D, Beyotime) for 0, 3, or 6 h. FOXD1 transcript stability was assessed by measuring residual mRNA levels at each timepoint.

### ChIP-quantitative PCR

The ChIP assay was conducted with ChIP Assay Kit (Beyotime) using antibodies against Flag (#20543-1-AP, Proteintech) or an IgG isotype control (#ab172730, Abcam). Briefly, Flag-FOXD1-transfected HNE3 and C666-1 cells were fixed with formaldehyde at a final concentration of 0.75%, followed by glycine treatment to terminate cross-linking. The fixed cells were then subjected to ultrasonication to shear chromatin into fragments. The resulting DNA fragments were incubated overnight with antibody-conjugated magnetic A/G Dynabeads. After immunoprecipitation, the enriched DNA was purified and analyzed by quantitative PCR to determine the enrichment abundance of the NAT10 promoter regions.

### Luciferase reporter assay

The NAT10 promoter fragments containing either the predicted binding sequence (WT) or its mutated version (MUT) were cloned into the pGL3-Basic reporter vector (Miaoling Plasmid) to generate wild-type (WT-NAT10) and mutant (MUT-NAT10) constructs. Each reporter construct (WT-NAT10 or MUT-NAT10) was introduced into HNE3 and C666-1 cells together with si-FOXD1 or si-NC and the pRL-TK *Renilla* internal control (Promega, Milano, Italy). Following a 48-hour incubation period, luminescence signals were detected using the Luciferase Assay system (Promega).

### Statistical analysis

Statistical analyses were performed using Mann-Whitney *U* test or unpaired *t*-test for two-group comparisons. For multi-group analyses, one-way or two-way ANOVA with Tukey’s or Sidak’s post hoc correction was applied. Results were presented as mean ± SD, with statistical significance defined as *P* < 0.05. Pearson’s correlation analysis was employed to evaluate expression relationships, and Kaplan-Meier survival curves with log-rank tests were utilized for prognostic assessment.

## Results

### High FOXD1 expression correlates with NPC cell growth, invasion, apoptosis, and sphere formation potential in vitro

**Fig. 1:**
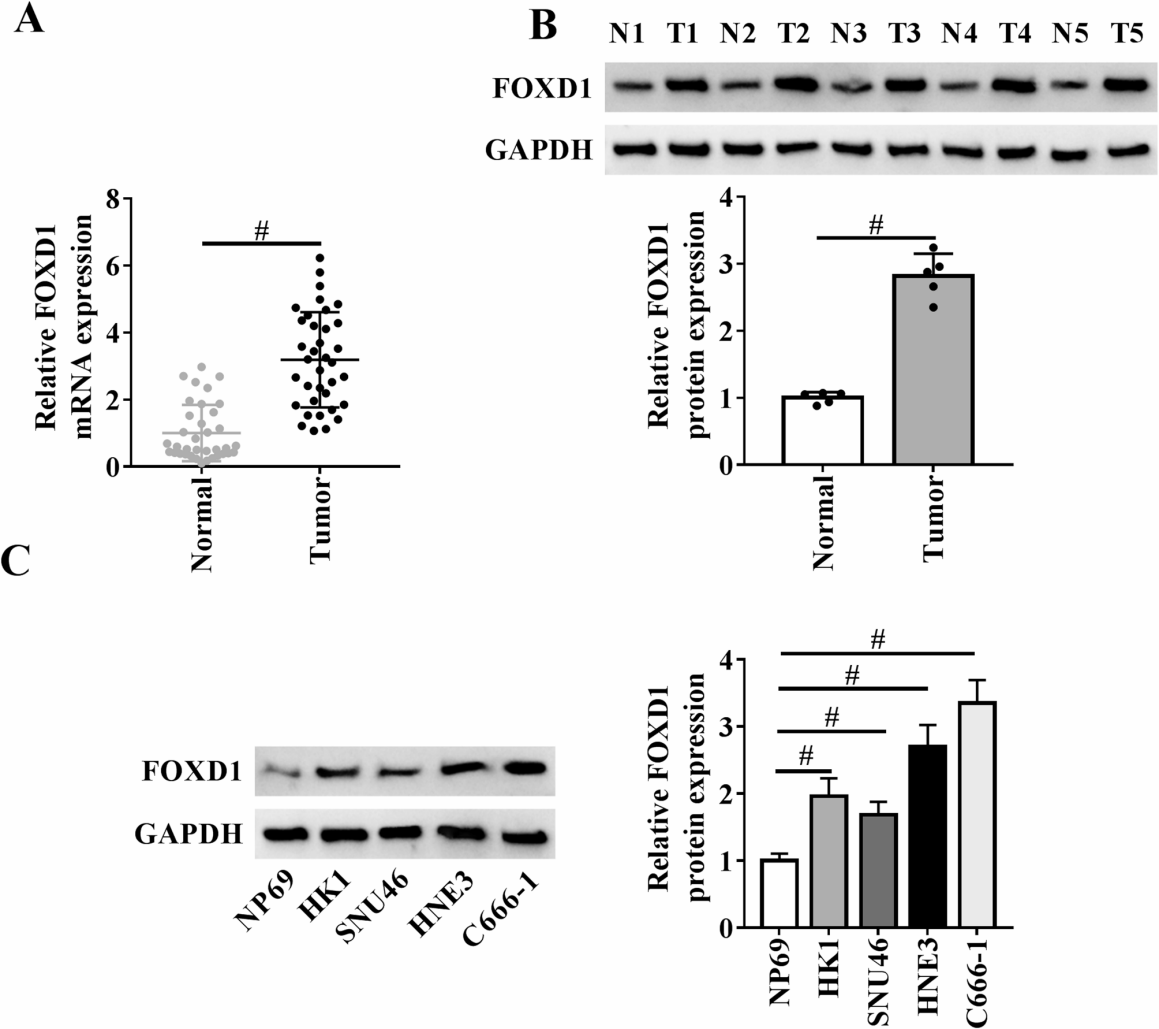
FOXD1 expression is increased in NPC. (**A**) Quantitative PCR of clinical NPC samples (*n* = 36) and matched non-tumor counterparts (*n* = 36) showing increase in FOXD1 mRNA levels in cancer tissues. (**B**) Immunoblot assay of clinical NPC samples (*n* = 5) and adjacent non-tumor tissues (*n* = 5) depicting upregulation in FOXD1 protein expression in NPC cancer tissues. (**C**) Immunoblot assay in lysates from NPC cell lines (HK1, SNU46, HNE3, and C666-1) and non-tumor NP69 cells and quantification of FOXD1 protein expression relative to the loading control GAPDH. ^#^*P* < 0.05

To demonstrate the involvement of FOXD1 in NPC, this study first tested its expression profile in a cohort of clinical NPC samples. Upregulation of FOXD1 in these clinical cancer tissues was validated by quantitative PCR and immunoblot assays compared to the matched normal counterparts (Fig. 1A and B). Moreover, using the median FOXD1 expression as a cutoff, Kaplan-Meier analysis revealed significantly worse overall survival in patients with high FOXD1 expression relative to those with low expression (Supplementary Fig. 1A). Consistently, high protein levels of FOXD1 were observed in NPC cell lines compared with NP69 control cells (Fig. 1C).

**Fig. 2:**
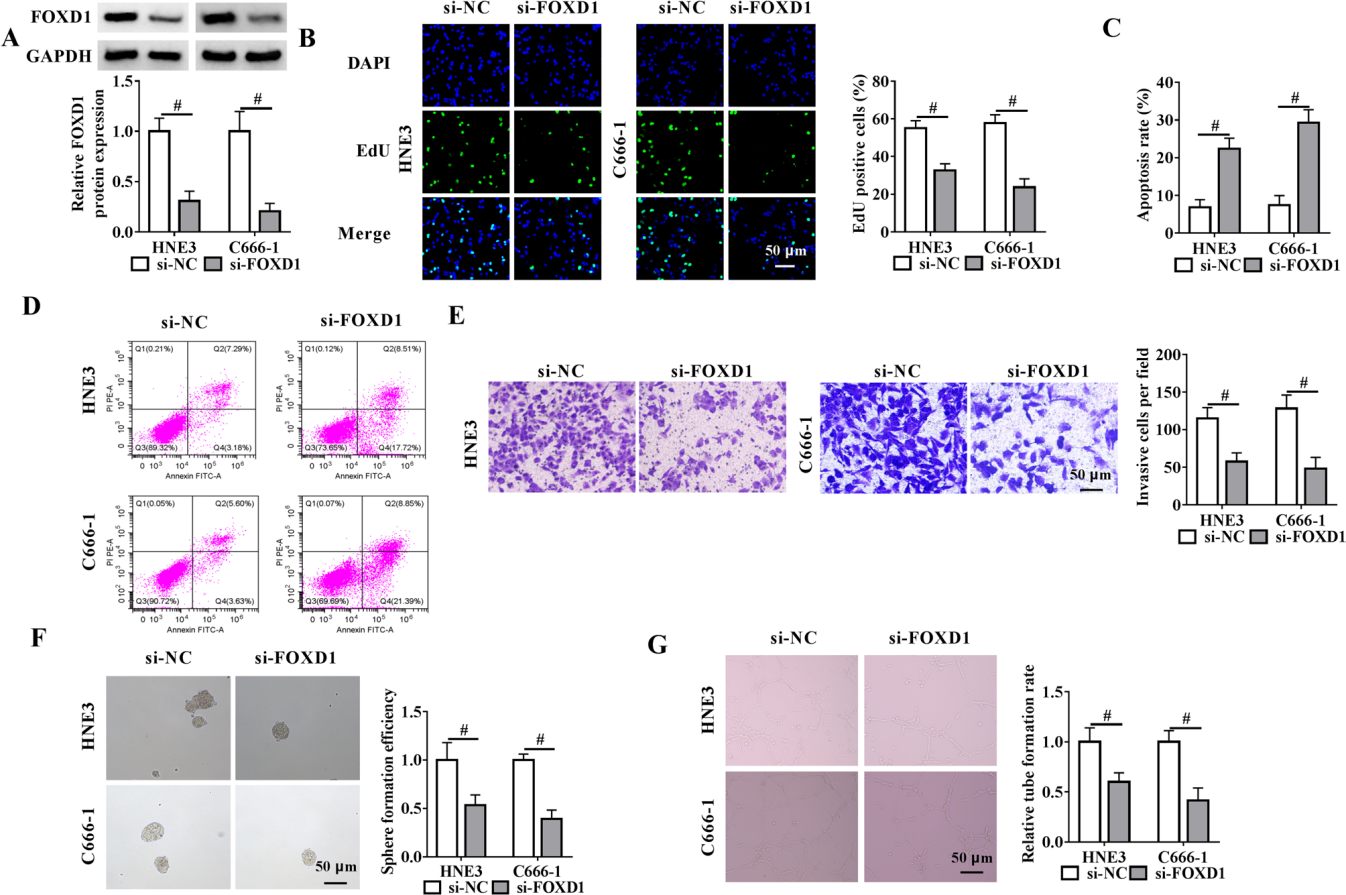
FOXD1 depletion weakens NPC cell growth, invasion, sphere formation ability, while promoting cell apoptosis and impairing HUVEC tube formation. (**A**) Immunoblot assay in lysates from si-FOXD1- or si-NC-transfected HNE3 and C666-1 cells after 48 h transfection and quantification of FOXD1 protein expression. (**B**) EdU incorporation assay with si-FOXD1- or si-NC-transfected cells and assessment of the percentage of EdU positive cells. (**C** and **D**) Flow cytometry with si-FOXD1- or si-NC-transfected cells and determination of the apoptosis rate. (**E**) Transwell assay with si-FOXD1- or si-NC-transfected cells showing the invasive ability alteration following FOXD1 depletion. (**F**) Sphere formation assay with cells transfected as indicated depicting cell stemness changes after FOXD1 silencing. (**G**) Tube formation assay with HUVECs treated with the conditioned medium of si-FOXD1- or si-NC-transfected cells and quantification of tube formation rate. Scale bars: 50 μm. ^#^*P* < 0.05

Functional analysis of FOXD1 was then performed by creating depletion cell lines using si-FOXD1 or si-FOXD1#2 in HNE3 and C666-1 NPC cell lines, which were chosen based on their relatively high endogenous FOXD1 expression levels (Fig. 1C). The silencing efficiency of FOXD1 at the protein level in both cell lines was shown in Fig. 2A and Supplementary Fig. 2A. FOXD1 silencing led to the reduction in the percentage of EdU positive cells (Fig. 2B and Supplementary Fig. 2B) and the increase in the rate of apoptosis (Fig. 2C and D and Supplementary Fig. 2C and 2D) relative to si-NC scramble. By contrast, FOXD1 depletion also reduced cell invasive potential in HNE3 and C666-1 cells (Fig. 2E and Supplementary Fig. 2E). Consistently, knockdown of FOXD1 resulted in a clear diminishment of cellular sphere formation efficiency (Fig. 2F and Supplementary Fig. 2F). Additionally, FOXD1 knockdown significantly repressed cell cycle progression of HNE3 NPC cells (Supplementary Fig. 3A). To assess if FOXD1 affects NPC angiogenesis, HUVECs were treated with the conditioned medium of FOXD1-depleted NPC cells and quantified their tube formation ability. A significant decrease in the rate of tube formation was observed in HUVECs incubated with FOXD1-depleted NPC cells compared with si-NC controls (Fig. 2G and Supplementary Fig. 2G). All these data demonstrate the pro-tumorigenic activity of FOXD1 in NPC.

### NAT10 mediates the ac4C acetylation of FOXD1 mRNA

**Fig. 3:**
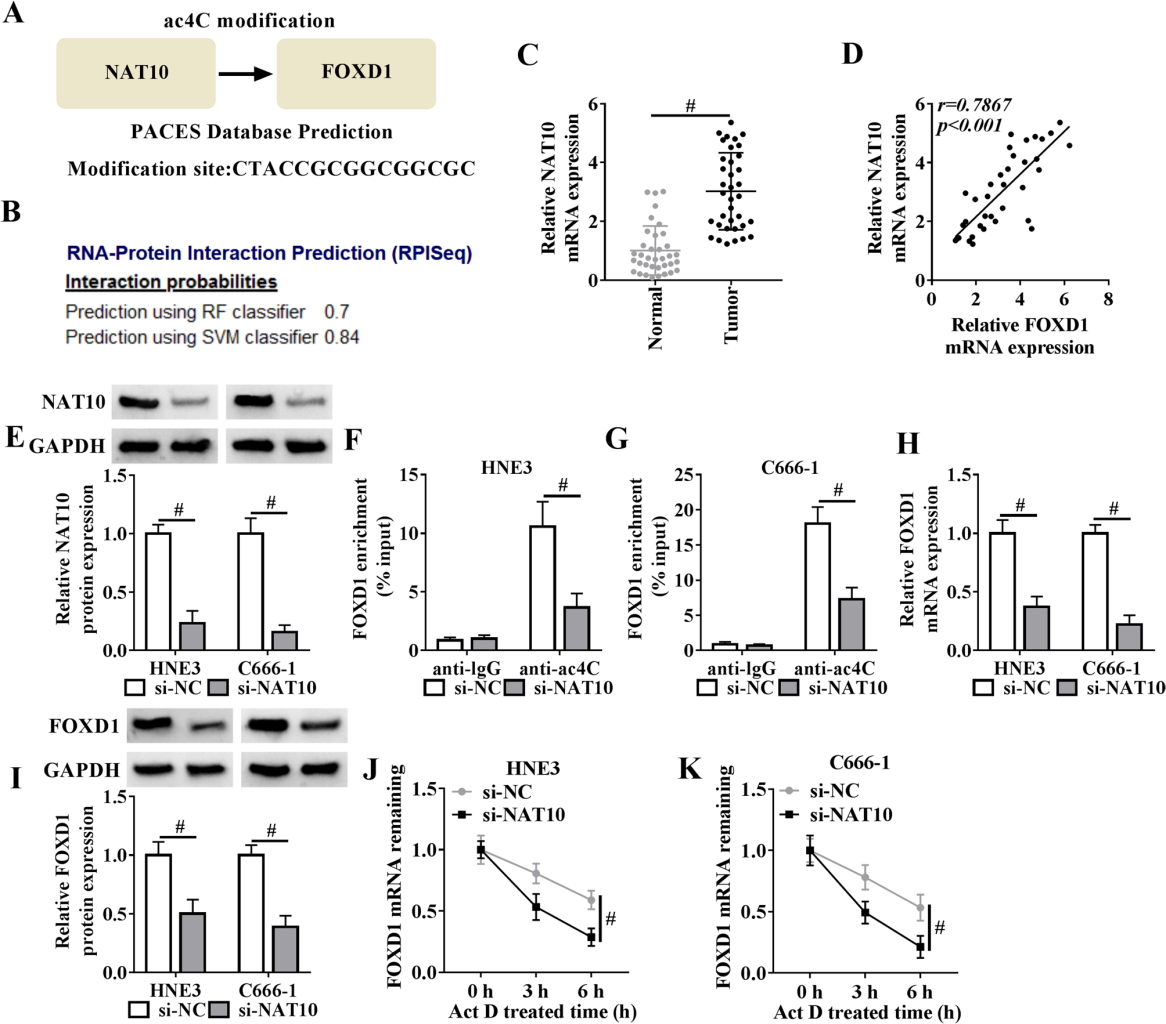
NAT10 stabilizes FOXD1 mRNA by mediating its ac4C modification. (**A**) Schematic of the interaction between NAT10 and FOXD1 and the putative ac4C modification site within FOXD1 mRNA predicted by PACES web-based platform. (**B**) The potential interaction between NAT10 and FOXD1 mRNA predicted using RPISeq computational method. (**C**) Quantitative PCR of clinical NPC samples (*n* = 36) and matched non-tumor counterparts (*n* = 36) showing increase in NAT10 mRNA levels in cancer tissues. (**D**) Expression association between NAT10 and FOXD1 in clinical NPC samples (*n* = 36). (**E**) Immunoblot analysis in lysates from si-NAT10- or si-NC-transfected HNE3 and C666-1 cells after 48 h transfection and quantification of NAT10 protein levels relative to GAPDH. (**F** and **G**) RIP assay in lysates from transfected cells using anti-ac4C or anti-IgG antibody followed by quantitative PCR analysis and binding quantification for FOXD1 mRNA enrichment levels. (**H**) Quantitative PCR in lysates from transfected cells showing the reduction in FOXD1 mRNA expression following NAT10 depletion. (**I**) Immunoblot analysis in lysates from si-NAT10- or si-NC-transfected HNE3 and C666-1 cells after 48 h transfection showing the decrease in FOXD1 protein expression after NAT10 depletion. (**J** and **K**) Transfected cells were exposed to Act D for 0, 3, and 6 h, followed by quantification of FOXD1 mRNA by quantitative PCR. ^#^*P* < 0.05

Recent studies reveal that aberrant N4-acetylcytidine (ac4C) modification, catalyzed by NAT10, drives cancer progression by stabilizing oncogenic mRNAs [15, 16]. Intriguingly, bioinformatic analysis using the PACES web-based platform identified a putative ac4C modification site (CTACCGCGGCGGCGC) within FOXD1 mRNA (Fig. 3A), while RPISeq computational modeling further indicated a potential interaction between NAT10 and FOXD1 mRNA (Fig. 3B). NAT10 expression was significantly increased in NPC tissues compared with normal controls (Fig. 3C). Importantly, a positive and significant expression association between NAT10 and FOXD1 was found in these NPC tissues (Fig. 3D). Furthermore, when patients were stratified into low and high NAT10 expression groups based on median expression levels, those with high NAT10 expression demonstrated significantly shorter overall survival compared to the low-expression group (Supplementary Fig. 1B). Since NAT10 is a well-established ac4C writer in promoting cancer progression, our study proposed that it may mechanistically regulate FOXD1 ac4C modification in NPC cells. To address this hypothesis, NAT10 was silenced with a specific siRNA pool (si-NAT10) in HNE3 and C666-1 NPC cells. Immunoblot analysis confirmed the efficiency of the knockdown (Fig. 3E). RIP experiments were then performed using a specific antibody to ac4C and the following quantitative PCR. These experiments showed the binding of FOXD1 mRNA to the ac4C-associating immunoprecipitates in HNE3 and C666-1 cells (Fig. 3F and G), confirming the indeed occurrence of ac4C acetylation of FOXD1 mRNA. Importantly, NAT10 silencing significantly decreased the levels of ac4C-modified FOXD1 mRNA (Fig. 3F and G). Furthermore, depletion of NAT10 led to a striking decrease in FOXD1 mRNA and protein levels in the two NPC cell lines (Fig. 3H and I). Under transcriptional blockade with actinomycin D (Act D), NAT10 knockdown significantly reduced the half-life period of FOXD1 mRNA (Fig. 3J and K). Collectively, these findings establish that NAT10 mediates FOXD1 mRNA ac4C modification to stabilize this transcript.

### Ectopic FOXD1 expression reverses NAT10 depletion-driven phenotypic alterations in NPC cells

**Fig. 4:**
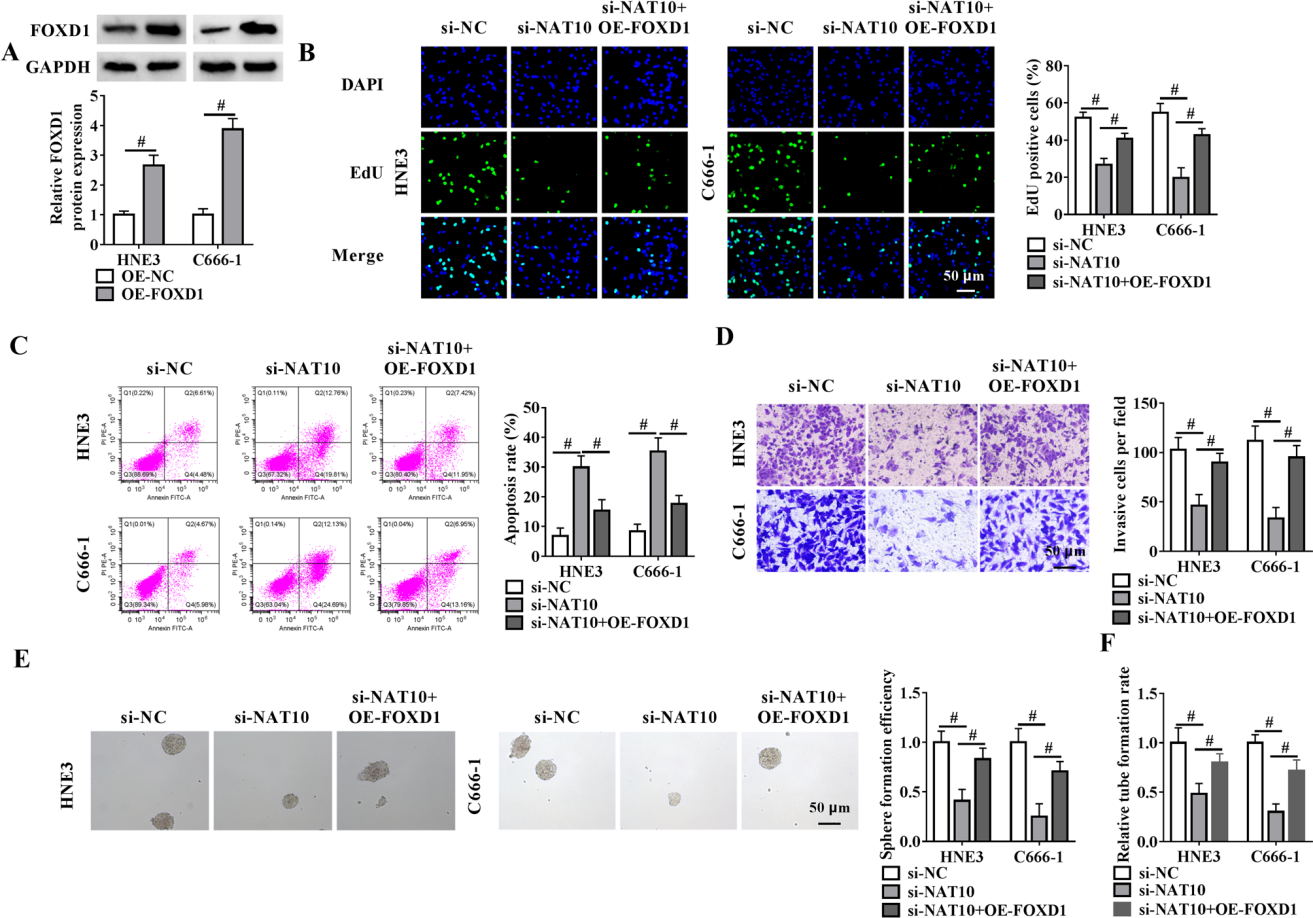
FOXD1 upregulation counteracts NAT10 depletion-driven phenotypic alterations in NPC cells. (**A**) Immunoblot analysis in lysates from OE-FOXD1- or OE-NC-transfected HNE3 and C666-1 cells after 48 h transfection and quantification of FOXD1 protein expression relative to GAPDH. (**B**-**E**) HNE3 and C666-1 cells were subjected to transfection with si-NC, si-NAT10, or si-NAT10 + OE-FOXD1 and checked for the percentage of EdU positive cells by EdU incorporation assay (**B**), the apoptosis rate by flow cytometric analysis (**C**), the number of invasive cells by transwell assay (**D**), and the sphere formation efficiency (**E**). Scale bars: 50 μm. (**F**) Tube formation assay with HUVECs treated with the conditioned medium of cells transfected as indicated and quantification of tube formation rate. ^#^*P* < 0.05

Having identified NAT10 as an upstream regulator of FOXD1 expression, our study next examined whether NAT10 modulates NPC progression via FOXD1. Immunoblotting validated the upregulation efficiency of a FOXD1 expression construct (OE-FOXD1) in NPC cells (Fig. 4A). By contrast, NAT10 depletion resulted in a pronounced reduction in the percentage of EdU positive cells (Fig. 4B), a augmentation in the apoptosis rate (Fig. 4C), and a clear decrease in the number of invasive cells (Fig. 4D) in HNE3 and C666-1 cells, which could be markedly abolished by increased FOXD1 expression (Fig. 4B and D). Ectopic FOXD1 expression also diminished si-NAT10-driven repression in cell sphere formation efficiency (Fig. 4E). NAT10 silencing significantly impaired HUVEC tube formation, while this alteration was largely reversed by ectopic expression of FOXD1 (Fig. 4F). Additionally, NAT10 silencing strongly repressed cell cycle progression of HNE3 NPC cells (Supplementary Fig. 3B). These data together support the notion that NAT10 regulates NPC progression via FOXD1.

### FOXD1 is a transcriptional regulator of NAT10 in NPC cells

**Fig. 5:**
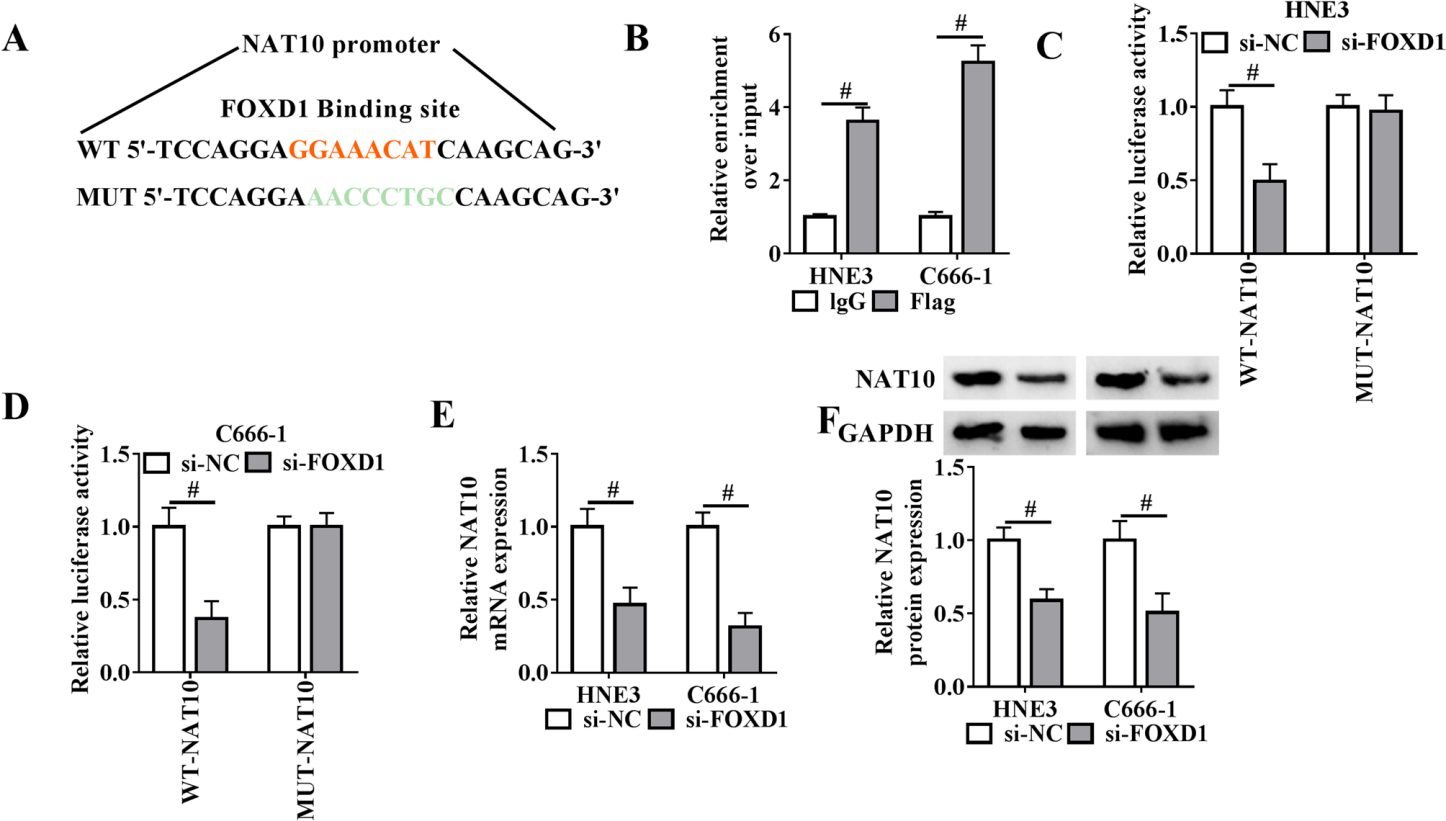
FOXD1 acts as a transcriptional activator of NAT10 in NPC cells. (**A**) The predicted binding site for FOXD1 within the NAT10 promoter region (-2000 bp to TSS) using JASPAR and the mutated site sequence. (**B**) ChIP assay in lysates from Flag-FOXD1-transfected HNE3 and C666-1 cells using anti-Flag or anti-IgG antibody followed by quantitative PCR for the enrichment levels of the NAT10 promoter fragments. (**C** and **D**) Luciferase reporter assay in cells after 48 h transfection with si-NC + WT-NAT10, si-NC + MUT-NAT10, si-FOXD1 + WT-NAT10, or si-FOXD1 + WT-NAT10. (**E**) Quantitative PCR in lysates from si-FOXD1- or si-NC-transfected cells showing the reduction in NAT10 mRNA expression following FOXD1 depletion. (**F**) Immunoblot analysis in lysates from si-FOXD1- or si-NC-transfected HNE3 and C666-1 cells after 48 h transfection showing the decrease in NAT10 protein expression after FOXD1 silencing. ^#^*P* < 0.05

Little is still known regarding the transcriptional control of NAT10. FOXD1 is a crucial transcription factor that orchestrates oncogenic processes through its regulatory role in gene expression [9]. Of particular interest, the JASPAR online algorithm predicted the binding of FOXD1 on the NAT10 promoter region (-2000 bp to TSS) (Fig. 5A). To demonstrate the binding, ChIP assay was carried out by transfecting a Flag-FOXD1 construct into NPC cells. The fragments of the NAT10 promoter region were substantially bound by Flag-FOXD1 (Fig. 5B). To investigate the control of FOXD1 in NAT10 transcription, luciferase experiments were performed by creating wild-type NAT10 promoter reporter (WT-NAT10) or mutated reporter construct (MUT-NAT10). Depletion of FOXD1 by si-FOXD1 led to reduced luciferase activity in cells harboring the WT-NAT10 construct (Fig. 5C and D). However, when the FOXD1-binding site was disrupted (MUT-NAT10), the suppressive effect of si-FOXD1 was no longer observed (Fig. 5C and D). Moreover, FOXD1 knockdown resulted in a significant reduction of NAT10 expression at both mRNA and protein levels in HNE3 and C666-1 NPC cells (Fig. 5E and F). Thus, these findings indicate that FOXD1 activities NAT10 transcription to upregulate NAT10 expression in NPC cells.

### FOXD1 depletion induces phenotypic alterations in NPC cells through the reduction of NAT10

**Fig. 6:**
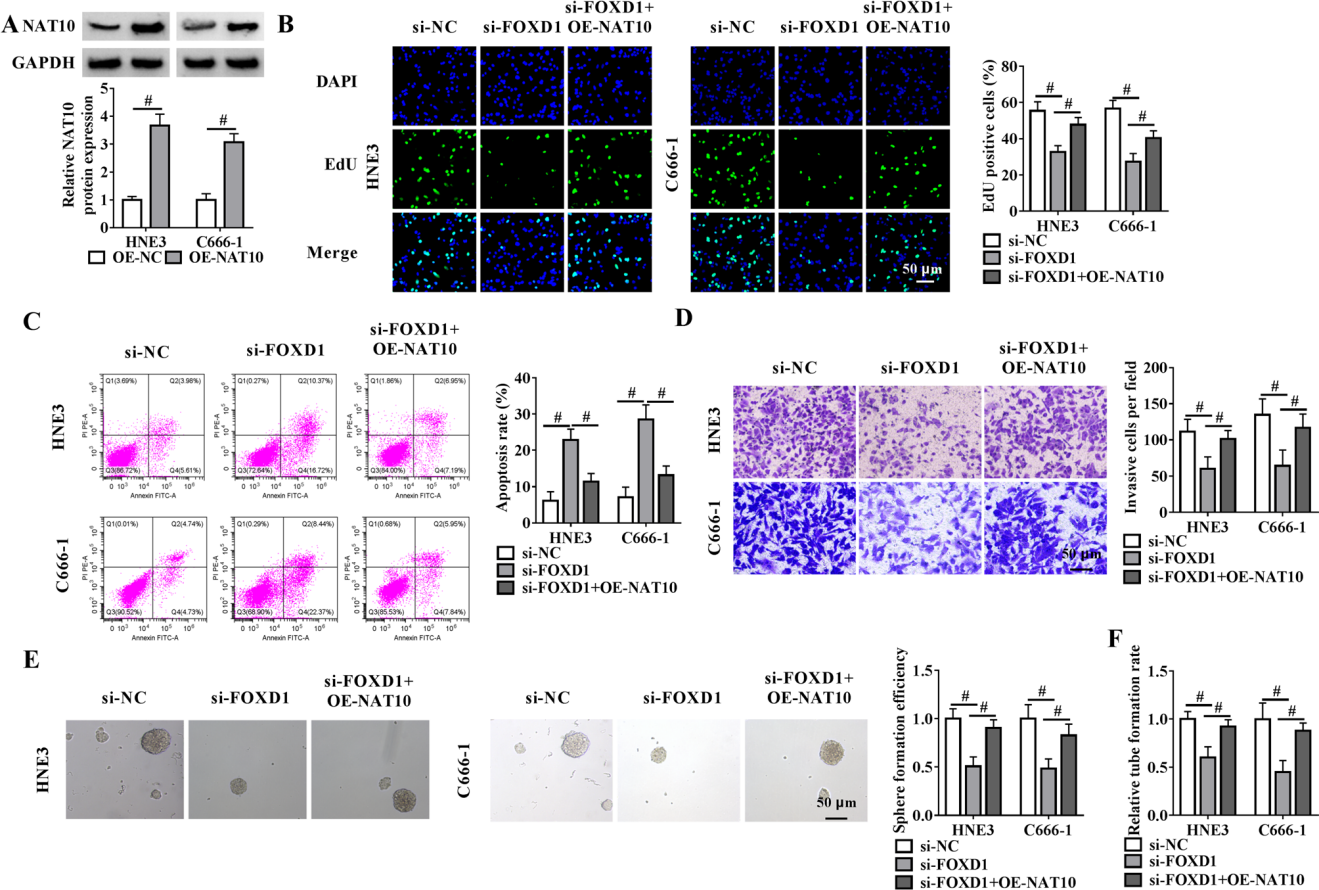
NAT10 reconstitution reverses FOXD1 depletion-driven phenotypic alterations in NPC cells. (**A**) Immunoblot analysis in lysates from OE-NAT10- or OE-NC-transfected HNE3 and C666-1 cells after 48 h transfection and quantification of NAT10 protein expression relative to GAPDH. (**B**-**E**) HNE3 and C666-1 cells were introduced with si-NC, si-FOXD1, or si-FOXD1 + OE-NAT10, followed by determination of the percentage of EdU positive cells by EdU incorporation assay (**B**), the apoptosis rate by flow cytometric analysis (**C**), the number of invasive cells by transwell assay (**D**), and the sphere formation efficiency (**E**). Scale bars: 50 μm. (**F**) Tube formation assay with HUVECs treated with the conditioned medium of cells transfected as indicated and quantification of tube formation rate. ^#^*P* < 0.05

To test whether FOXD1 regulates NPC progression through NAT10, NAT10 rescue experiments were performed by transfecting FOXD1-depleted cells with a NAT10 overexpression vector (OE-NAT10). Figure 6A revealed the efficiency of the increase at NAT10 protein levels in both cell lines. In contrast, FOXD1 knockdown significantly decreased the proportion of EdU-positive cells (Fig. 6B), increased apoptosis (Fig. 6C), and reduced cellular invasion (Fig. 6D) in both HNE3 and C666-1 cell lines. Notably, these effects were substantially reversed by ectopic NAT10 expression (Fig. 6B and D). Furthermore, increased NAT10 expression attenuated the inhibitory effect of FOXD1 depletion on NPC cell sphere formation (Fig. 6E). Silencing of FOXD1 significantly attenuated angiogenic capacity, as evidenced by impaired HUVEC tube formation, while NAT10 reconstitution effectively reversed this inhibitory phenotype (Fig. 6F). Taken together, these results establish NAT10 as a critical downstream effector of FOXD1 in NPC progression.

### FOXD1 reconstitution rescues NAT10 depletion-mediated tumor growth inhibition in vivo

**Fig. 7:**
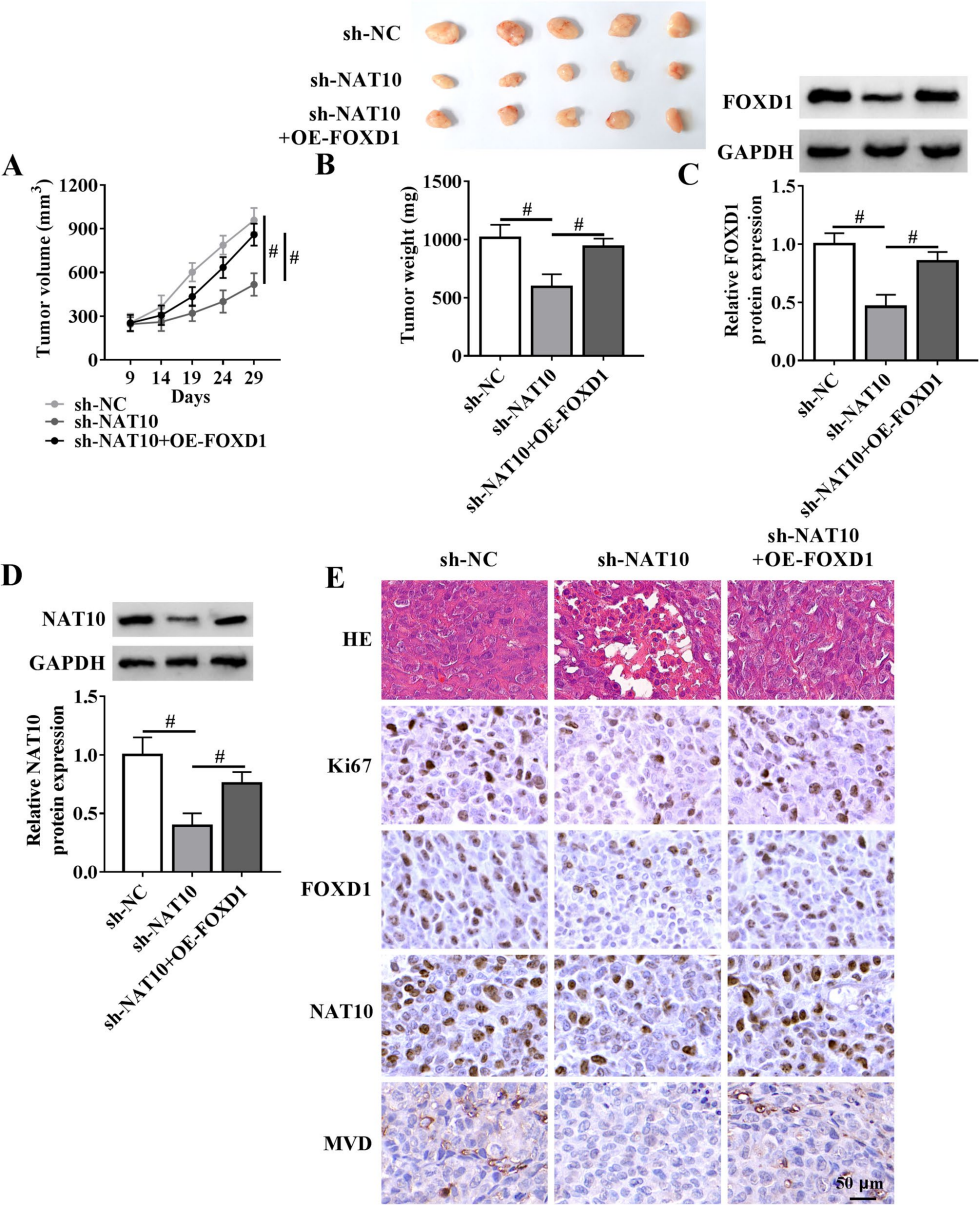
NAT10 knockdown inhibits NPC xenograft growth by reducing FOXD1 levels. For xenograft studies, 2 × 10^6^ lentivirus-transduced C666-1 cells (sh-NC or sh-NAT10) were implanted subcutaneously in the right flank of nude mice. Intratumoral OE-FOXD1 delivery was performed upon reaching a tumor volume of ~ 100 mm^3^. Each group included five mice. 29 days later, C666-1 NPC xenografts were harvested. (**A**) Tumor volume progression curves in C666-1 xenograft-bearing mice. (**B**) Tumor morphology and mean tumor weight of C666-1 NPC xenografts. (**C** and **D**) Immunoblot analysis in lysates from C666-1 NPC xenografts and quantification of FOXD1 and NAT10 protein expression. (**E**) H&E staining and immunohistochemistry analysis of C666-1 NPC xenografts. Scale bar: 50 μm. ^#^*P* < 0.05

Having established that ectopic FOXD1 expression reverses NAT10 depletion-driven phenotypic changes in vitro, this study finally determined whether FOXD1 reconstitution could also rescue the impact of NAT10 depletion on subcutaneous xenograft growth in vivo. For this purpose, nude mice received subcutaneous injections of C666-1 NPC cells transduced with either sh-NC or sh-NAT10 lentivirus in the right flank, followed by intratumoral delivery of OE-FOXD1. As expected, sh-NAT10-transduced cells produced significantly smaller tumors than sh-NC controls (Fig. 7A and B). However, sh-NAT10-mediated tumor growth inhibition was rescued by OE-FOXD1 administration (Fig. 7A and B). Immunoblot and immunohistochemistry analyses showed reduced expression of FOXD1 and NAT10 in sh-NAT10 xenografts compared to sh-NC controls, while OE-FOXD1 increased their levels (Fig. 7C and E). Consistent with these observations in tumor growth, sh-NAT10-derived xenografts showed reduced number of Ki67 positive cells compared with sh-NC controls (Fig. 7E), supporting the suppression of NAT10 silencing on tumor growth. Furthermore, FOXD1 reconstitution increased the number of Ki67 positive cells in sh-NAT10-derived xenografts (Fig. 7E). Histopathological analysis (H&E staining) revealed that sh-NAT10-derived xenografts exhibited reduced cell density, abnormal tissue structure, and enlarged intercellular spaces relative to sh-NC controls (Fig. 7E), reinforcing NAT10 depletion-mediated tumor growth suppression. Strikingly, these histological changes were strongly reversed by FOXD1 reconstitution (Fig. 7E). Additionally, NAT10 silencing diminished tumor microvessel density (MVD) of C666-1 NPC xenografts, which could be reversed by FOXD1 reconstitution (Fig. 7E). All these data demonstrate that NAT10 depletion suppresses NPC xenograft growth by downregulating FOXD1.

## Discussion

Emerging evidence implicates ac4C modification as a pivotal epigenetic driver in cancer, where aberrant acetylation of oncogenic transcripts enhances mRNA stability, fostering tumor invasiveness and therapy resistance [15]. Meantime, transcription factor-mediated transcriptional reprogramming orchestrates cancer cell plasticity and tumor development [22]. Our study uncovers a reciprocal regulatory loop in NPC: NAT10-mediated ac4C acetylation stabilizes FOXD1 mRNA, in turn, FOXD1 transcriptionally activates NAT10 expression. This self-reinforcing loop amplifies oncogenic signaling through dual post-transcriptional and transcriptional mechanisms, revealing a new mechanism and offering potential targets for NPC treatment.

As a key transcription factor, FOXD1 exhibits pleiotropic oncogenic roles across malignancies by controlling gene expression [9]. In pancreatic cancer, FOXD1 contributes to cancer development by promoting SLC2A1 transcription [10]. In prostate cancer, FOXD1 knockdown inhibits cancer cell metastatic potential through its regulatory impact on β-catenin expression [23]. In NPC, FOXD1 enhances tumor development and gemcitabine resistance by facilitating mitophagy by activating BNIP3 transcription and expression [13]. Our study confirms the upregulation of FOXD1 in NPC, in line with this previous report [13]. Silencing FOXD1 hinders proliferation and invasiveness in HNE3 and C666-1 NPC cells, supporting its oncogenic activity in NPC [13]. Cancer stemness, characterized by self-renewal capacity and differentiation plasticity, constitutes a critical driver of tumor recurrence and therapeutic resistance [24]. FOXD1 has emerged as a key regulator of stemness in various carcinomas, including colorectal cancer, where it enhances cell stemness to induce chemotherapy resistance via β-catenin [25], and oral squamous cell carcinoma, where it sustains stem cell features by transcriptionally enhancing SNAI2 expression [26]. Our findings expand this paradigm to NPC, revealing the potential regulation of FOXD1 in NPC cell stemness, though the experimental testimony is insufficient and needs further investigation. Tumor angiogenesis is essential for nutrient supply and metastatic dissemination [27]. Emerging studies delineate FOXD1 as a critical mediator of tumor vasculature. In colorectal cancer, upregulated FOXD1 drives VEGF-A-dependent angiogenesis via CXCR2 [28]. FOXD1 stabilization promotes vasculogenic mimicry in glioma cells by activating DKK1 transcription [29]. Our study shows that FOXD1 depletion attenuates HUVEC tube formation, suggesting its crucial role in NPC angiogenesis. This study establishes FOXD1 as a multi-functional oncoprotein in NPC, orchestrating tumor growth, cancer stemness, and angiogenesis.

The acetyltransferase NAT10, a pivotal “writer” of ac4C modification, has emerged as a critical oncogenic regulator across multiple malignancies partially by mediating mRNA stability. For example, NAT10 enhances the metastasis of esophageal cancer by stabilizing NOTCH3 mRNA via ac4C modification [15]. NAT10 mediates ac4C modification of oncogenes (BCL9L, SOX4, and AKT1) in bladder cancer to enhance their mRNA stability, thereby promoting cancer cell malignant behaviors and stem-cell-like properties [16]. Under hypoxia, HIF-1α-induced NAT10 stabilizes SEPT9 mRNA via ac4C modification, forming a NAT10/SEPT9/HIF-1α feedback loop that drives glycolysis addiction in gastric cancer [18]. In NPC, NAT10 can stabilize immunosuppressive transcripts (CEBPG/DDX5/HLTF) via ac4C modification, upregulating HMGB1 to impair T-cell function [19]. NAT10 confers sorafenib resistance in NPC by ac4C-mediated stabilization of SLC7A11 mRNA, suppressing ferroptosis [30].

In this study, our findings uncover a novel NAT10-dependent mechanism in NPC pathogenesis. First, NAT10 mediates ac4C acetylation of FOXD1 mRNA, stabilizing its transcript and amplifying FOXD1-driven oncogenic effects, including enhanced NPC cell proliferation, sphere formation potential, and HUVEC tube formation. Second, our data identify a reciprocal regulatory loop wherein FOXD1 transcriptionally activates NAT10 expression, creating a self-reinforcing circuit that perpetuates malignant progression. This mutual amplification mirrors feedback loops observed in other cancers, such as the NAT10/SEPT9/HIF-1α axis in gastric cancer glycolysis [18] and the NAT10/KIF23/GSK-3β axis in colorectal cancer progression [31], underscoring the critical role of NAT10 in sustaining oncogenic signaling networks. Our findings reveal the FOXD1/NAT10 axis as a previously unrecognized driver of NPC progression. This reciprocal loop not only amplifies ac4C-dependent mRNA stabilization but also integrates transcriptional and post-transcriptional regulation, offering a dual therapeutic vulnerability. Targeting this axis could disrupt both NAT10’s enzymatic activity and FOXD1’s transcriptional output, potentially overcoming resistance mechanisms seen in single-target approaches. In future work, validating NAT10/FOXD1 co-expression in large-scale NPC cohorts and exploring synergistic therapies combining NAT10 inhibitors with anti-angiogenic agents or immunotherapies are warranted.

However, our study has several limitations. First, the mechanistic exploration was confined to two NPC cell lines, which may not fully represent the heterogeneity of clinical tumors. Second, this study mainly focused on in vitro and xenograft models, and further studies are needed to confirm our findings in more diverse preclinical models. Third, while in vivo experiments confirmed tumor growth inhibition, the specific contributions of NAT10/FOXD1 cascade to metastasis or microenvironmental crosstalk remain unexplored. Moreover, the translational relevance of targeting this axis in human patients remains unvalidated, as our in vivo experiments relied solely on xenograft models without clinical correlation to NAT10/FOXD1 co-expression patterns in NPC cohorts. Additionally, immunohistochemical validation of FOXD1 expression in clinical NPC cancer specimens could not be obtained due to limited availability of suitable tissues samples, with plans to incorporate these analyses in future investigations when additional specimens become accessible.

In summary, our study elucidates a previously unrecognized regulatory mechanism in NPC pathogenesis, characterized by a mutually reinforcing FOXD1/NAT10 positive feedback loop that potently drives tumor progression. These findings not only deepen our understanding of the molecular underpinnings of NPC development but also reveal new therapeutic vulnerabilities for clinical intervention.

## Supplementary Information

Below is the link to the electronic supplementary material.

Supplementary Material 1

Supplementary Material 2

## Data Availability

No datasets were generated or analysed during the current study.
