# Supplementary material for: The FOXD1/NAT10 positive feedback loop drives nasopharyngeal carcinoma progression
Source: Hereditas. 2025 Sep 25;162:186. doi: 10.1186/s41065-025-00555-9 (PMC12465154; doi:10.1186/s41065-025-00555-9)
Supplement: Supplementary file 1 — Supplementary Material 1 [file 41065_2025_555_MOESM1_ESM.docx]

**
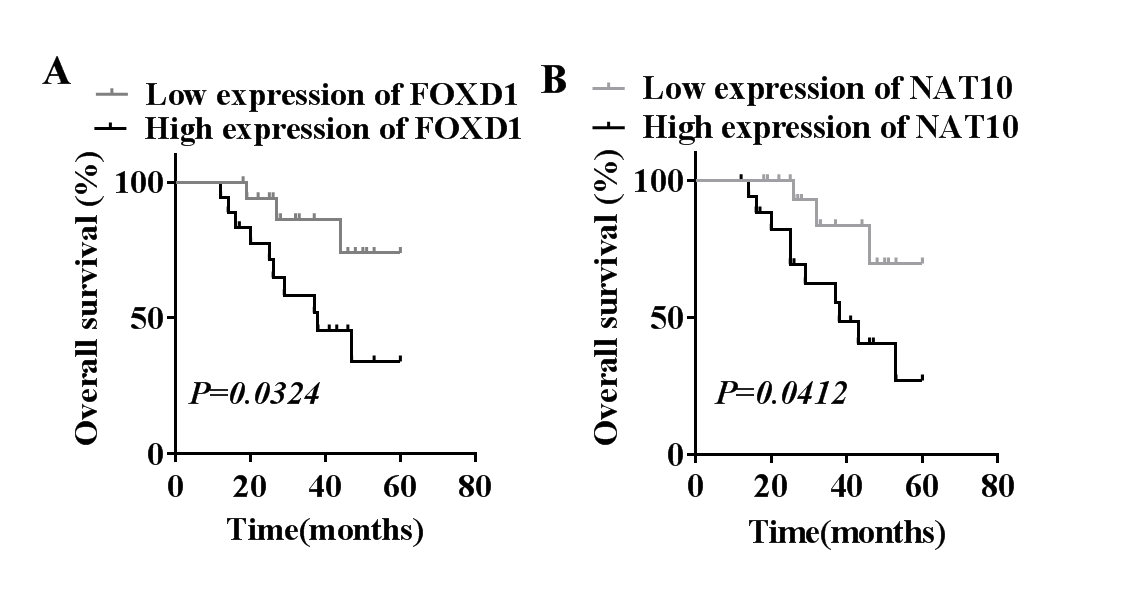
**

**Supplementary Figure 1.** Prognosis analysis of FOXD1 (A) and NAT10 (B) in clinical NPC patients.

**
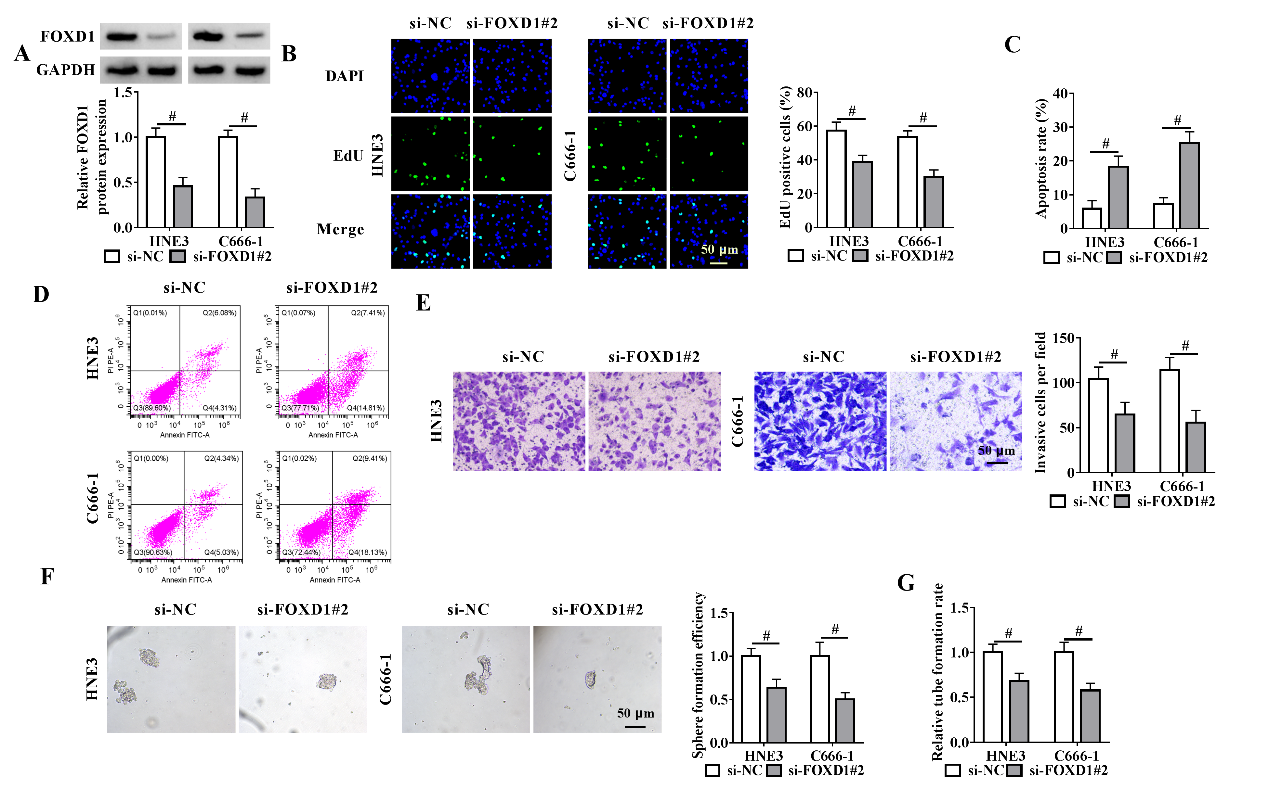
**

**Supplementary Figure 2.** Effects of FOXD1 depletion on NPC cell growth, invasion, sphere formation ability, apoptosis and HUVEC tube formation. (A) Immunoblot assay in lysates from si-FOXD1#2- or si-NC-transfected HNE3 and C666-1 cells after 48 h transfection and quantification of FOXD1 protein expression. (B) EdU incorporation assay with transfected cells. (C and D) Flow cytometry with transfected cells. (E) Transwell assay with transfected cells. (F) Sphere formation assay with cells transfected as indicated. (G) Tube formation assay with HUVECs treated with the conditioned medium of transfected cells and quantification of tube formation rate. Scale bars: 50 µm. ^#^*P*<0.05.

**
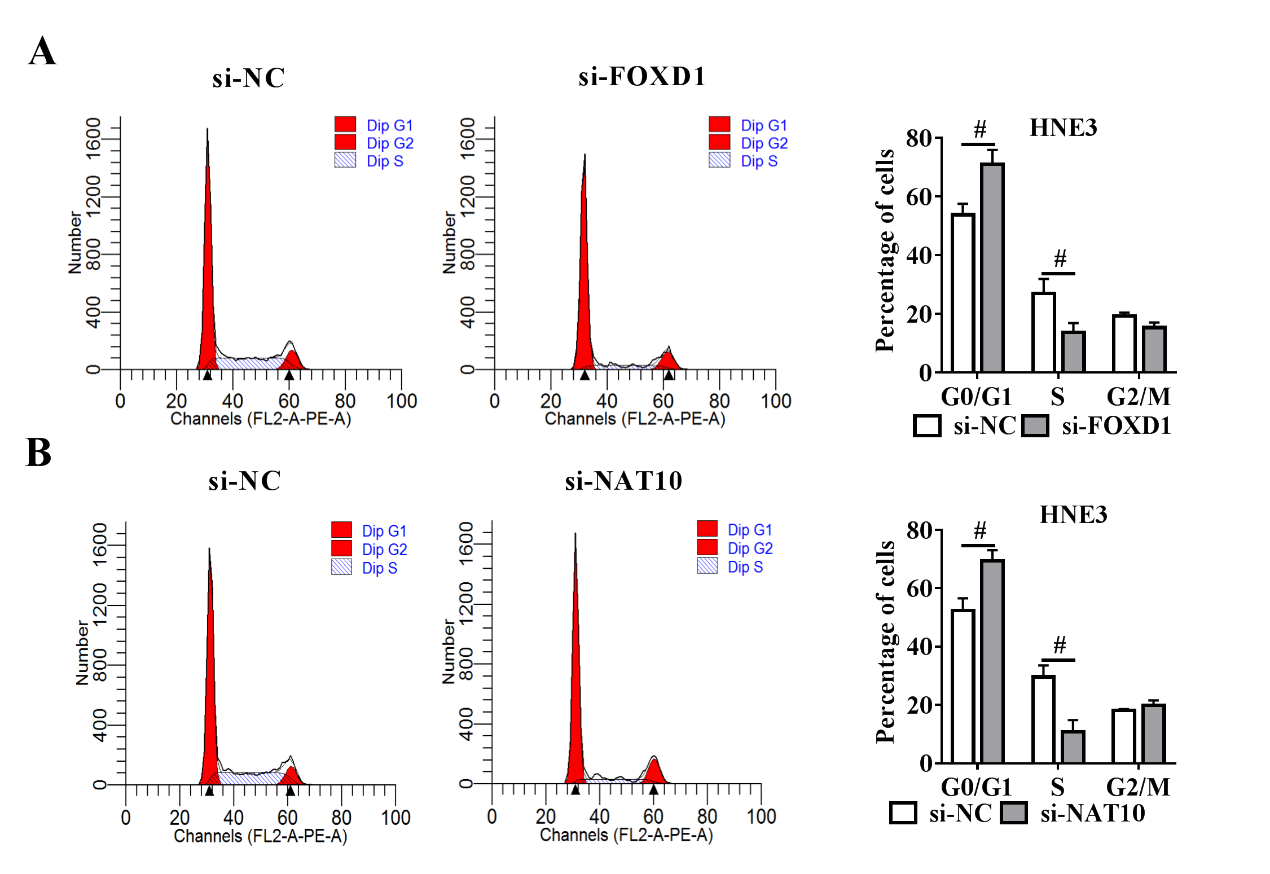
**

**Supplementary Figure 3.** Influence of si-FOXD1 or si-NAT10 on cell cycle progression. (A and B) Flow cytometry for cell cycle distribution in HNE3 cells transfected with si-NC, si-FOXD1, or si-NAT10. ^#^*P*<0.05.
