# Supplementary material for: The FOXD1/NAT10 positive feedback loop drives nasopharyngeal carcinoma progression
Source: Hereditas. 2025 Sep 25;162:186. doi: 10.1186/s41065-025-00555-9 (PMC12465154; doi:10.1186/s41065-025-00555-9)

The original western blots of Fig1

B

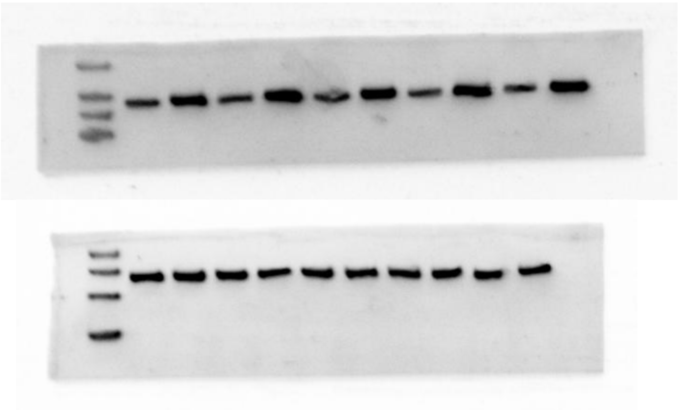

FOXD1

GAPDH

C

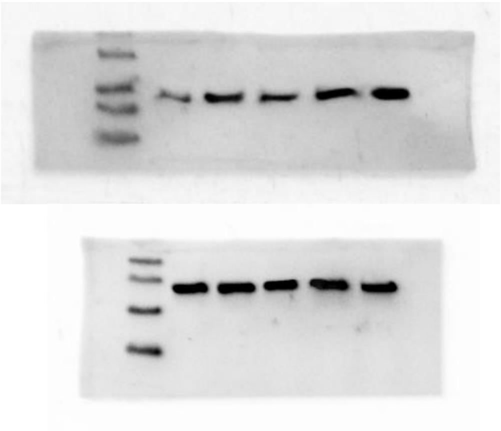

FOXD1

GAPDH

The original western blots of Fig2

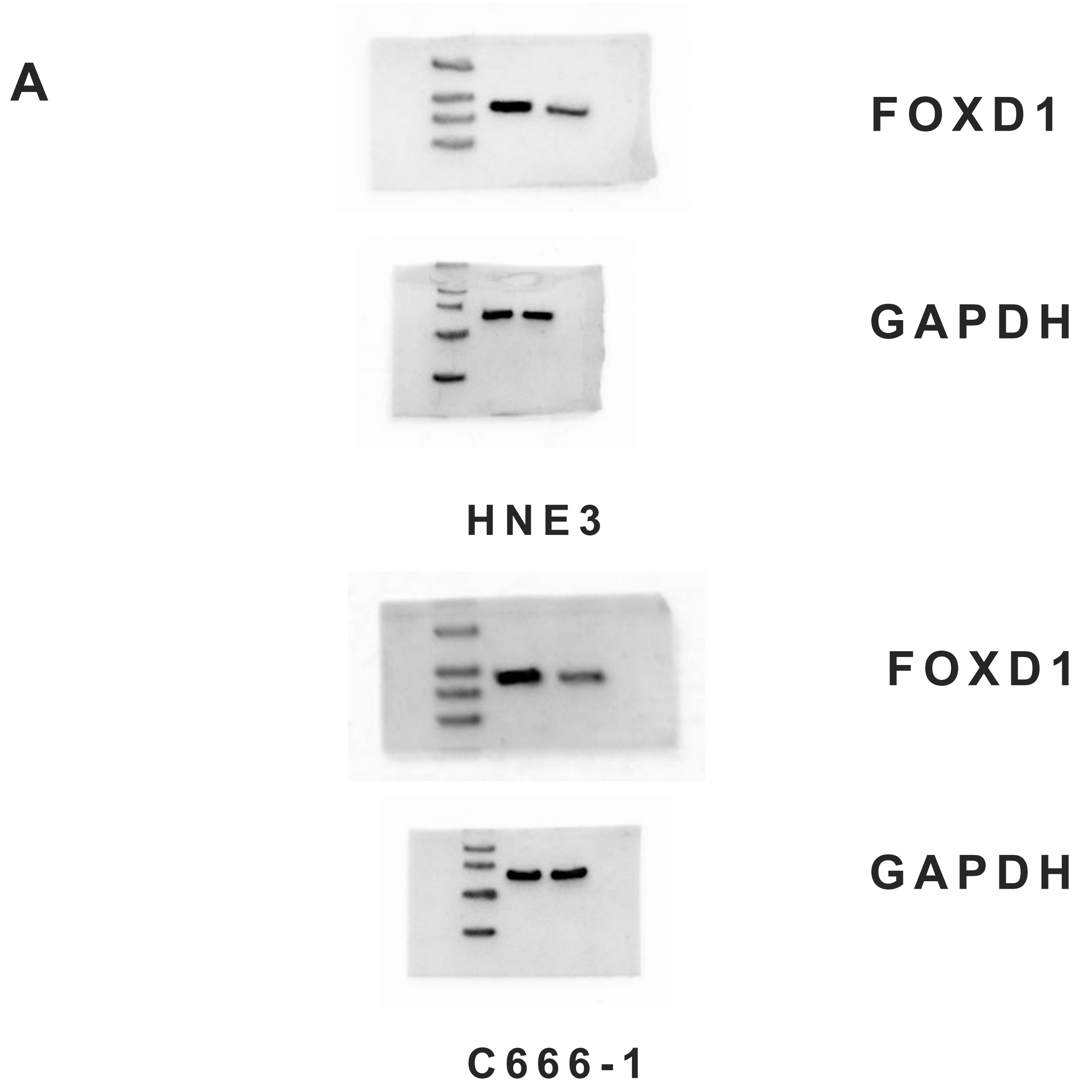

The original western blots of Fig3

C

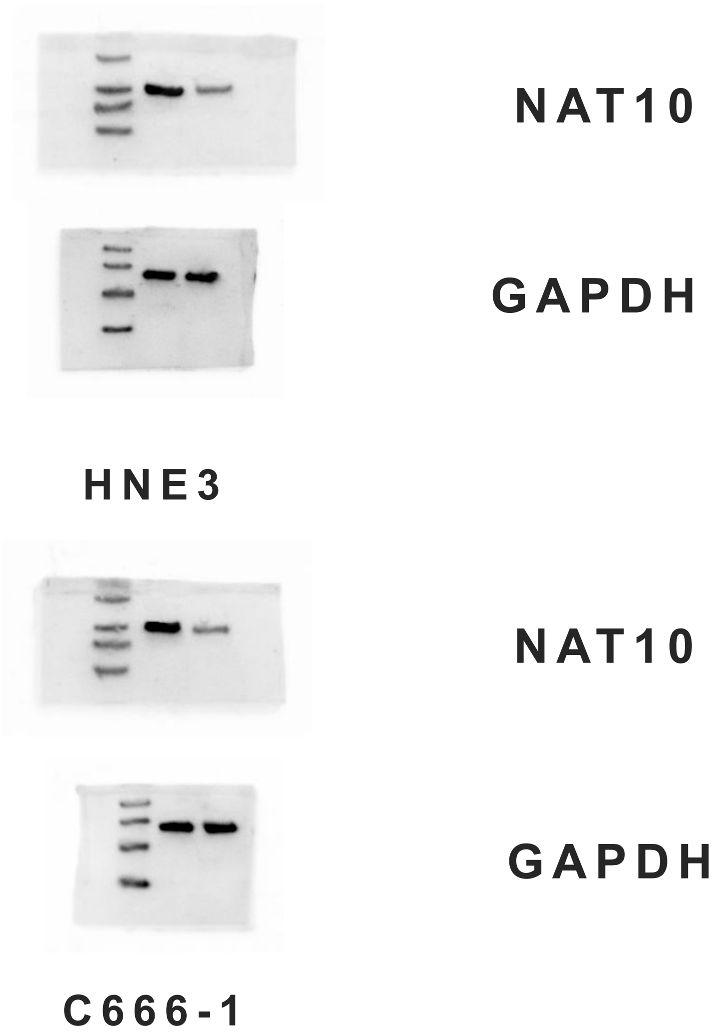

G

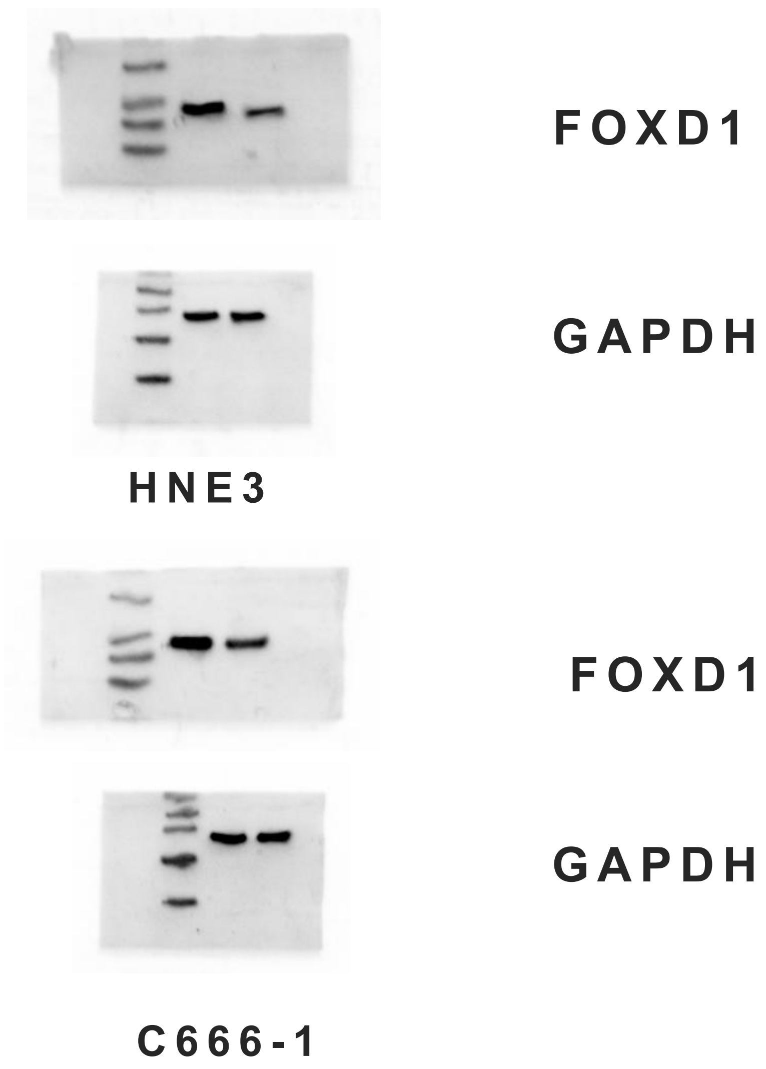

The original western blots of Fig4

A

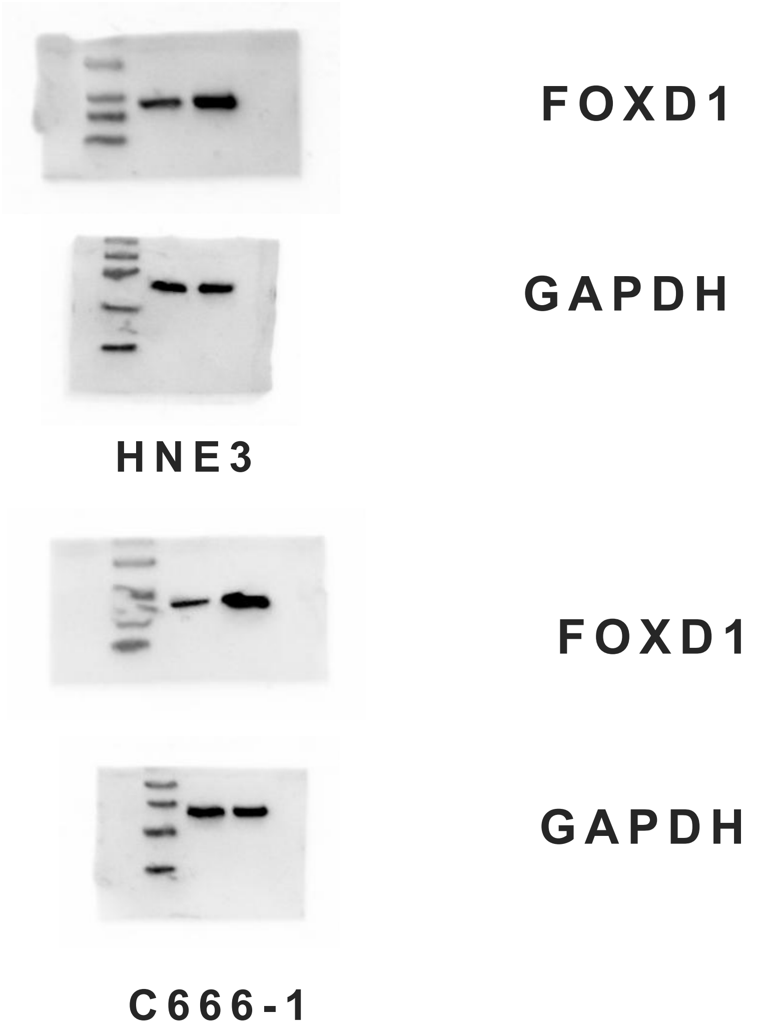

The original western blots of Fig5

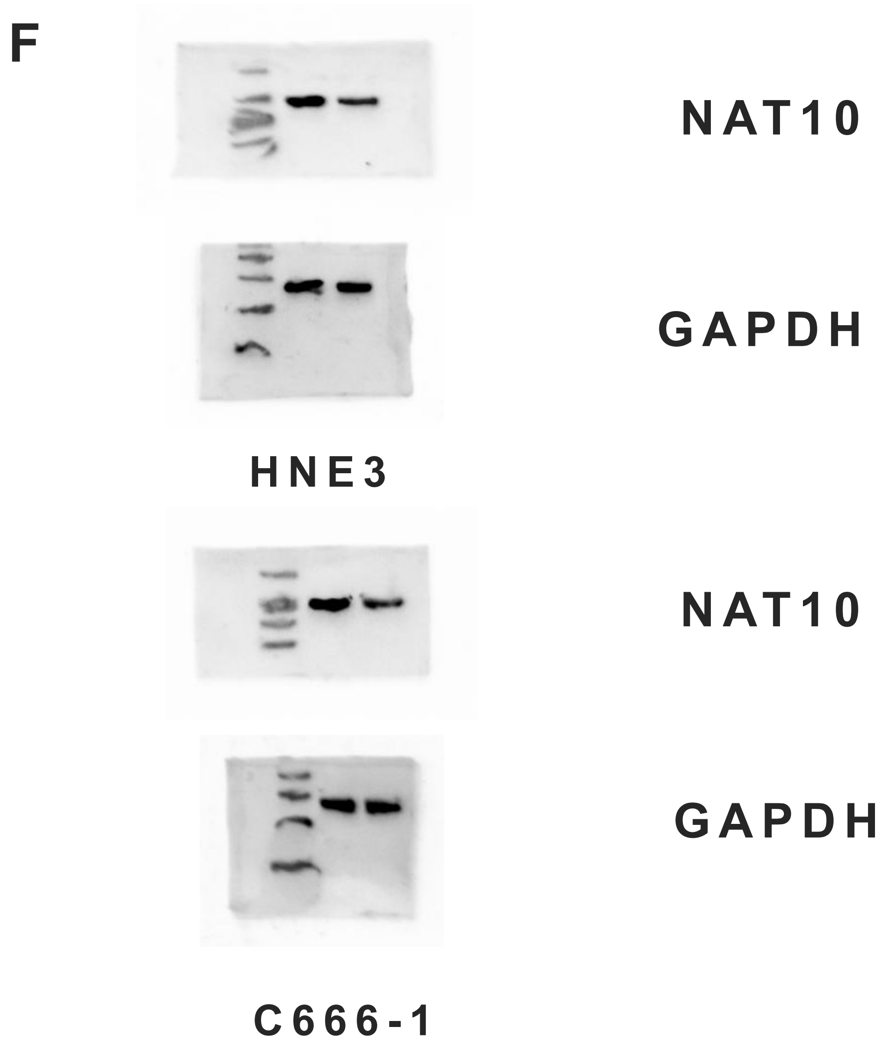

The original western blots of Fig6

A

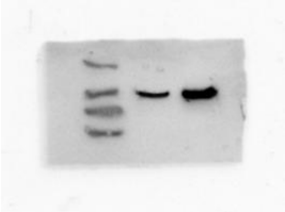

NAT10

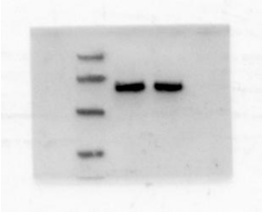

GAPDH

HNE3

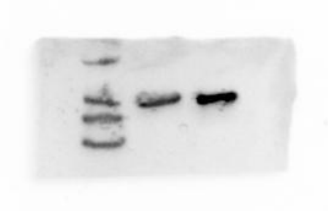

NAT10

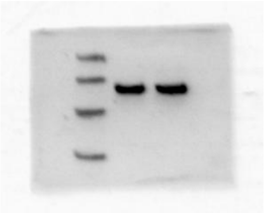

GAPDH

C666-1

The original western blots of Fig7

C

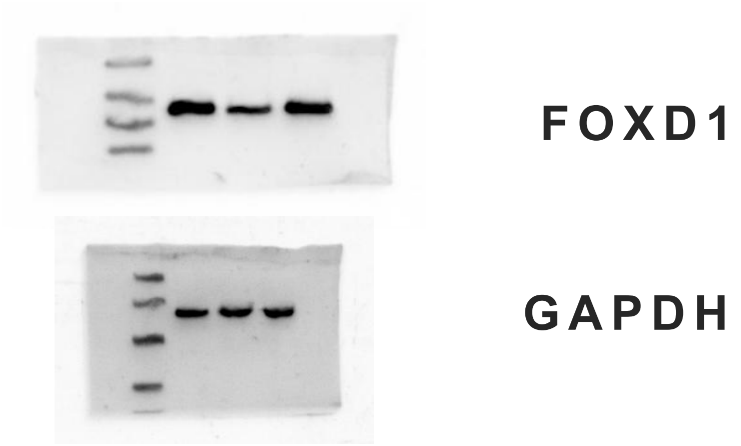

Supplement: Supplementary file 2 — Supplementary Material 2 [file 41065_2025_555_MOESM2_ESM.pdf]
